# Pinaverium bromide

**DOI:** 10.1107/S2414314624006539

**Published:** 2024-08-06

**Authors:** Yoann Rousselin, Alexandre Clavel

**Affiliations:** a Université de Bourgogne, ICMUB-UMR 6302, 9 avenue Alain Savary, 21000 Dijon, France; b M2I Salin, Route d’Arles, 13129 Salin-de-Giraud, France; Benemérita Universidad Autónoma de Puebla, México

**Keywords:** crystal structure, pinaverium bromide, morpholinium, anti-inflammatory properties, disorder

## Abstract

The structure of pinaverium bromide (systematic name: 4-[(2-bromo-4,5-di­meth­oxy­phen­yl)meth­yl]-4-{2-[2-(6,6-dimethyl-2-bi­cyclo­[3.1.1]hepta­nyl)eth­oxy]eth­yl}morpholin-4-ium bromide; C_26_H_41_Br_2_NO_4_), was determined at 110 K. It has monoclinic (*P*2_1_) symmetry. It is of inter­est with respect to its anti-inflammatory properties. The asymmetric unit contains two independent mol­ecules, one of which exhibits disorder of the bi­cyclo terminal group (occupancy factors: 0.78 and 0.22).

## Abstract 2

The title compound is a morpholinium derivative with the positive charge located on the N atom.

## Graphical abstract

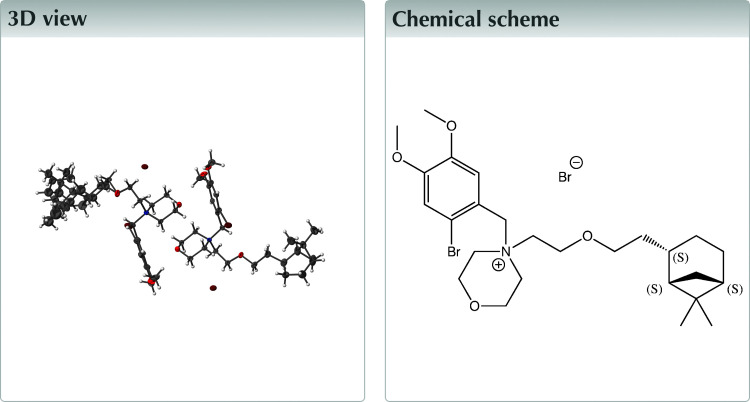

## Structure description

Pinaverium bromide is a medication used for functional gastrointestinal disorders. It belongs to a group of drugs called anti­spasmodics and acts as a calcium channel blocker, helping to restore the normal contraction process of the bowel (Zheng *et al.*, 2015 ▸). It is most effective when taken for a full course of treatment and is not designed for immediate symptom relief or sporadic, inter­mittent use (Wikipedia, 2023 ▸).

**Figure 1:**
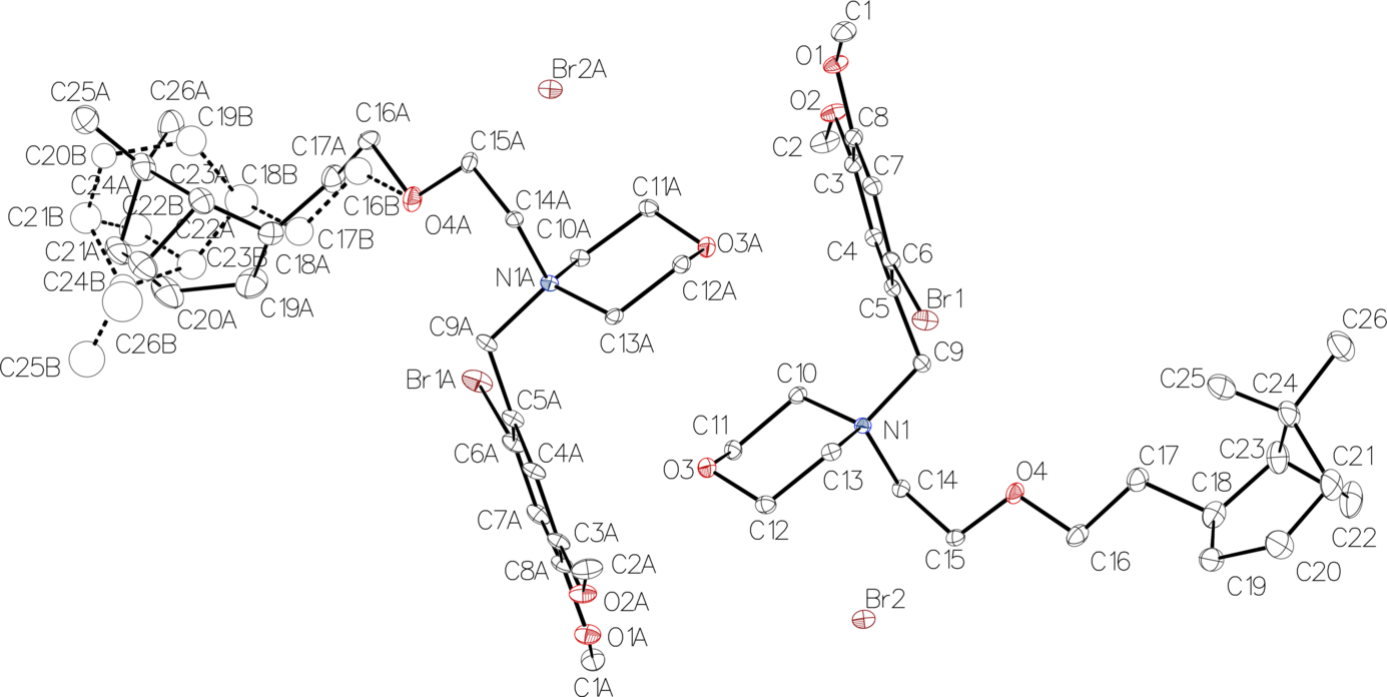
*ORTEP* view of the asymmetric unit of the title compound. Displacement ellipsoids are drawn at the 25% probability level. H atoms are omitted for clarity.

**Figure 2:**
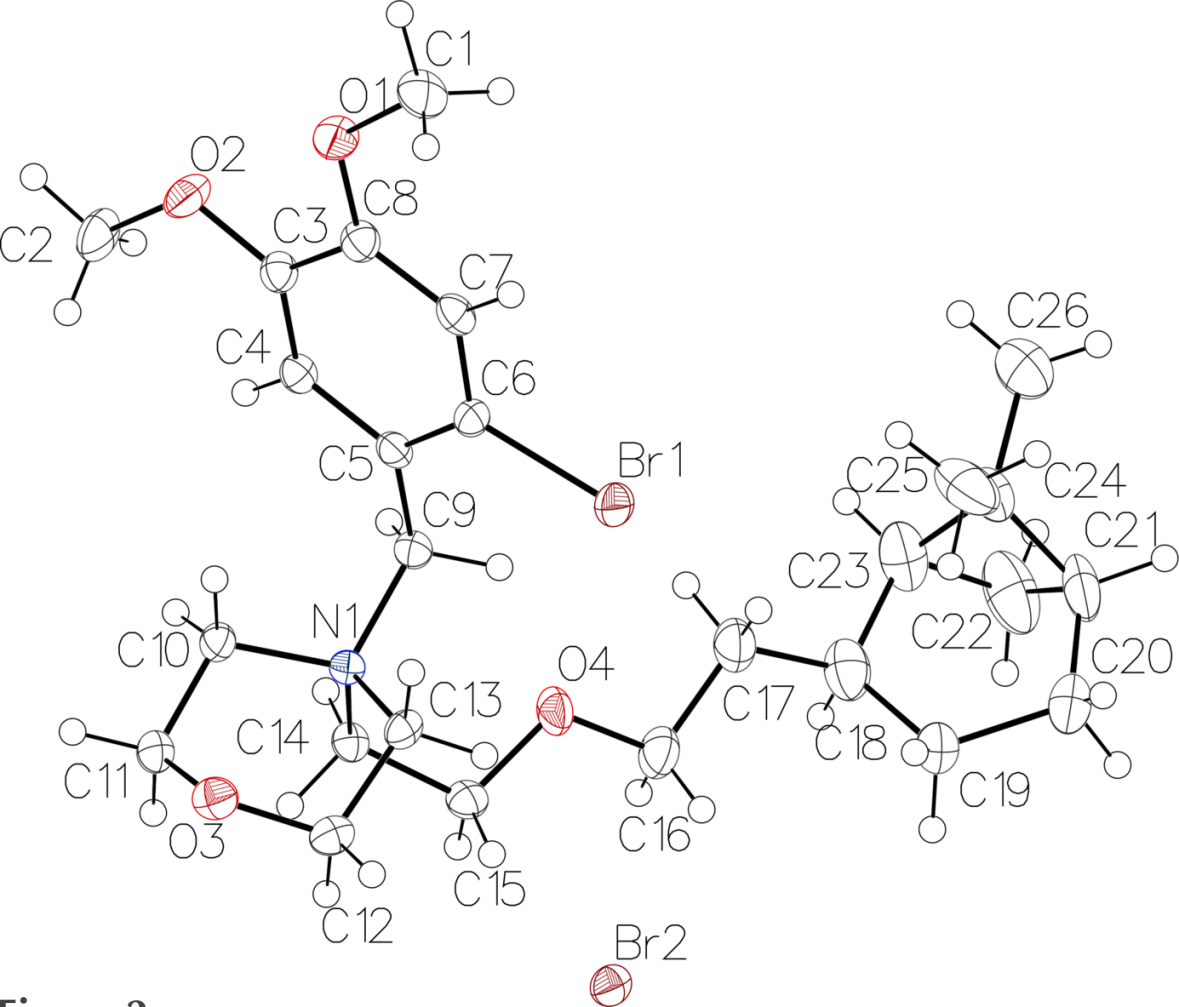
*ORTEP* view of the non-disordered mol­ecule, showing the absolute configuration for C18(*S*), C21(*S*) and C23(*S*).

The compound crystallizes with two mol­ecules per asymmetric unit (Fig. 1 ▸), one of which exhibits disorder of the di­methylbi­cyclo­heptane unit (fixed site occupancies: 0.78 and 0.22). The mol­ecule consists of a di­methylbi­cyclo­heptane linked by an eth­oxy­ethyl chain to a morpholinium group, which is itself linked to a bromo­dimeth­oxy­phenyl­methyl group. The species is present in the form of a cation, and the bromide counter-ion ensures neutrality. The charge is carried by nitro­gen, as indicated by the average C—N bond lengths of 1.528 (17) Å and the N—C—N angles of 109 (2)°. The ring puckering analysis (Cremer & Pople, 1975 ▸; Table 1 ▸) confirms the chair conformation of the morpholinium group and the half-boat conformation of the six-membered rings of the di­methylbi­cyclo­heptane groups in both mol­ecules. All di­methylbi­cyclo­hepta­nes have the same absolute configuration: *S*-C18, *S*-C21 and *S*-C23, as depicted in Fig. 2 ▸ for the non-disordered mol­ecule.

**Figure 3:**
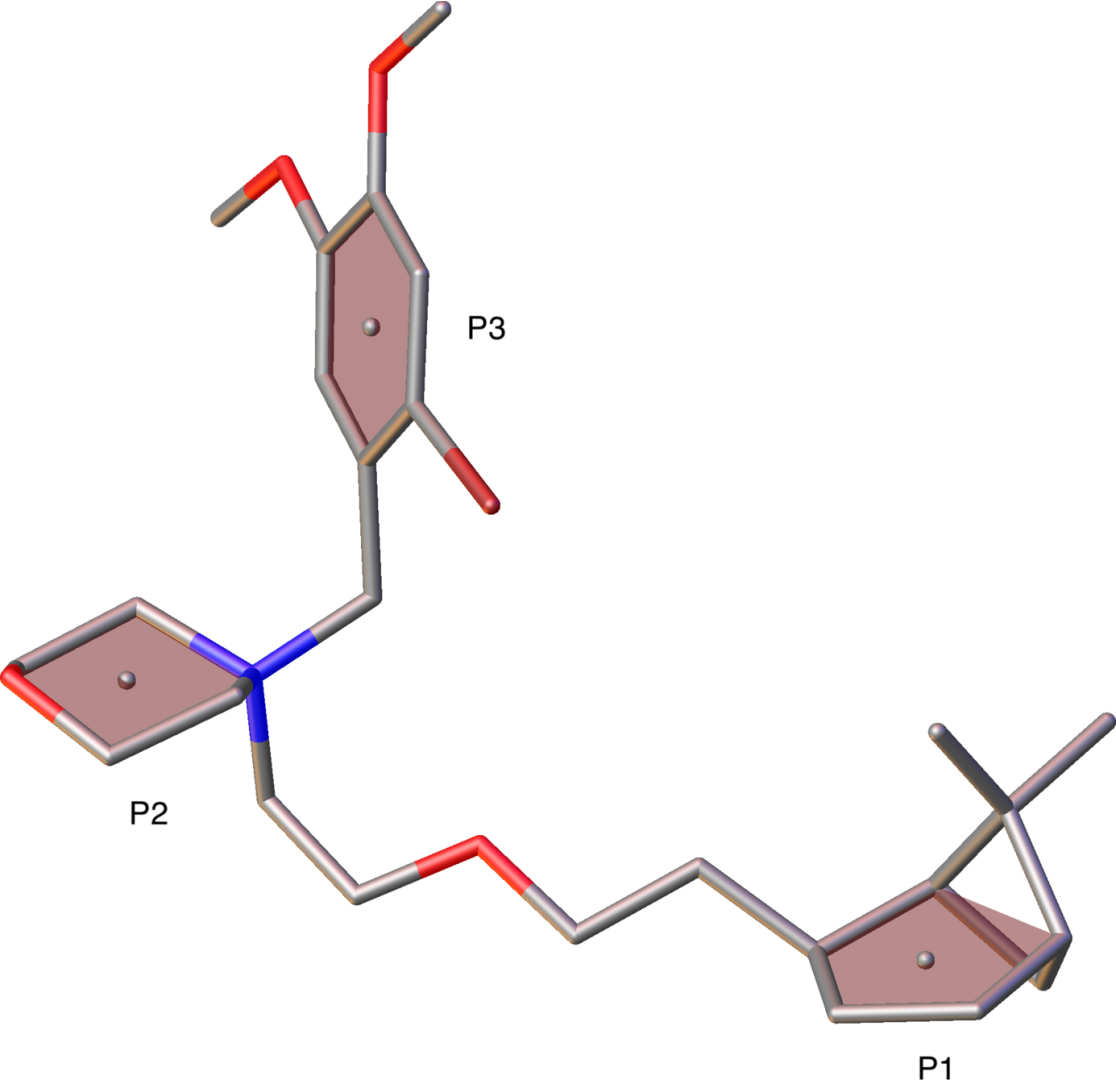
View of the three planes *P*1, *P*2 and *P*3. See definition in the main text.

**Figure 4:**
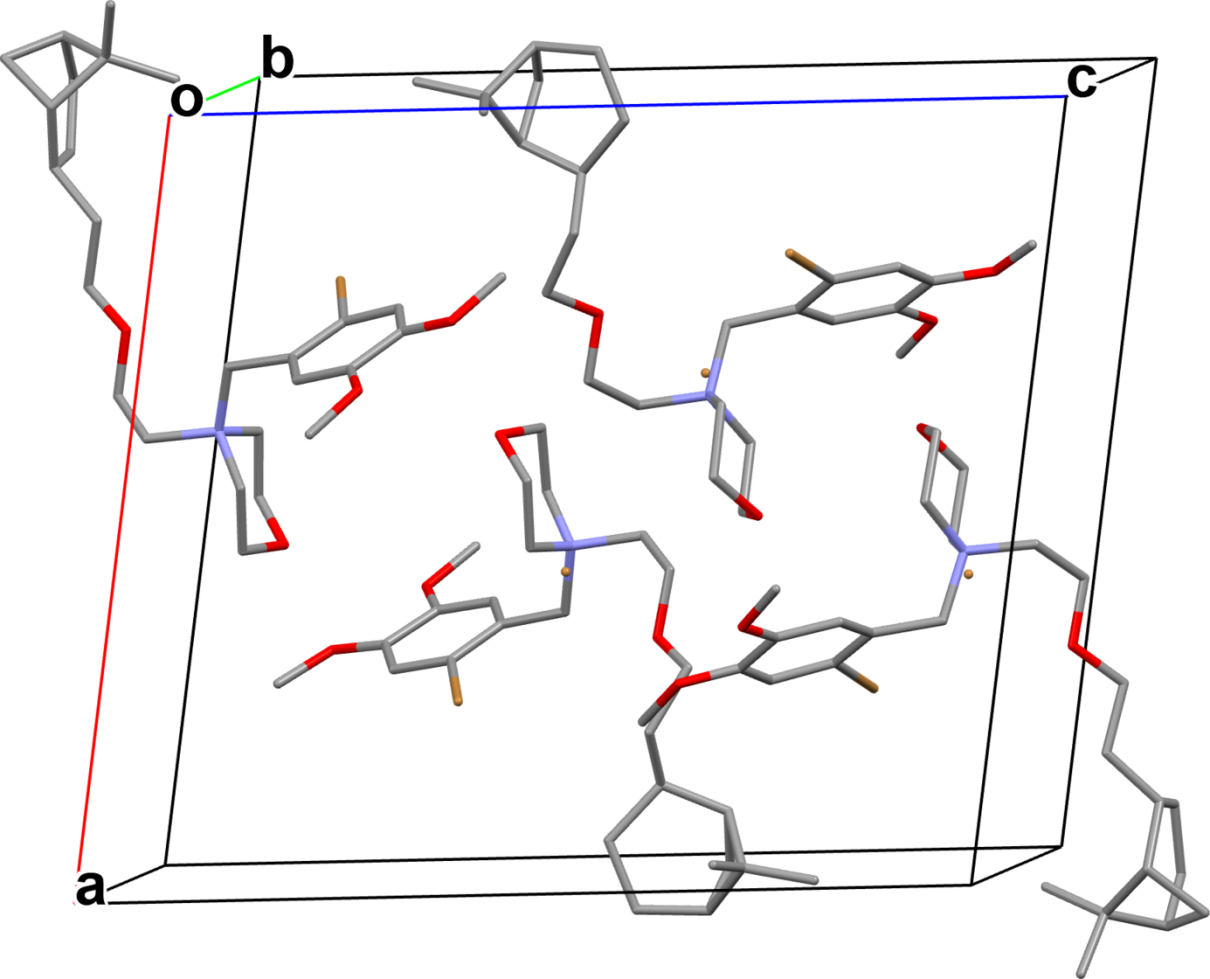
Crystal packing of the title compound, as viewed down the *b* axis. Disordered parts and H atoms are omitted for clarity.

If we define three planes *P*1, *P*2, and *P*3 (Fig. 3 ▸) corresponding respectively to the five atoms of the di­methylbi­cyclo­heptane (C18–C23), the plane formed by the six atoms of the morpholinium group (N1/O3/C10–C13), and the plane formed by the benzene ring of the bromo­dimeth­oxy­phenyl­methyl group (C3–C8), the angles between *P*1 and *P*2 vary in the two independent mol­ecules [15.23 (17), 88.1 (7) and 111.4 (3)°], whereas the angles between *P*2 and *P*3 remain close to a right angle [97.52 (13) and 90.84 (13)°]. The superposition of the mol­ecules, with the Automatic Mol­ecule Overlay feature of *Mercury* (Macrae *et al.*, 2020 ▸), results in an r.m.s. deviation of 1.332 Å and a maximum deviation of 3.743 Å. Overlaying the two mol­ecules shows that only the di­methylbi­cyclo­heptane part differs. In terms of the crystal packing, no specific inter­actions were found; the two mol­ecules are arranged head-to-tail in the unit cell (Fig. 4 ▸).

## Synthesis and crystallization

Pinaverium bromide was obtained by a two-step synthesis. A mixture of di­hydro­nopol, morpholino­chloro­ethane hydro­chloride and aqueous sodium hydroxide was stirred until completion of the reaction. The product was washed with water until the pH was 5–6, and the reaction mixture was then concentrated under reduced pressure. An equimolar amount of the isolated inter­mediate was then mixed with 1-bromo-2-bromo­methyl-4,5-di­meth­oxy­benzene in methyl ethyl ketone, and stirred at 333 K until completion of the reaction. The resulting solid precipitate was then washed several times with methyl ethyl ketone, giving pinaverium bromide as a white powder with an overall yield of 60%. Some colourless crystals were obtained by slow evaporation of a tri­fluoro­toluene solution.

## Refinement

Crystal data, data collection and structure refinement details are summarized in Table 2 ▸. One di­methylbi­cyclo­heptane group is disordered over two sites (parts *A* and *B* in Fig. 1 ▸), and their occupancies were fixed to 0.78 and 0.22. The minor part of the disorder (part *B*) was refined with isotropic C atoms. Moreover, displacement parameters in the disordered parts were restrained: in each part, C atoms were restrained to have the same displacement parameters, with standard deviation of 0.02 Å^2^, and rigid-bond hard restraints were applied, with standard deviations of 0.0001 Å^2^ for the 1,2 and 1,3 distances (*SIMU* and *RIGU* commands, respectively; Sheldrick, 2015*b* ▸). The absolute configuration was established by anomalous dispersion effects of Br sites.

## Supplementary Material

Crystal structure: contains datablock(s) I. DOI: 10.1107/S2414314624006539/bh4086sup1.cif

Structure factors: contains datablock(s) I. DOI: 10.1107/S2414314624006539/bh4086Isup2.hkl

Supporting information file. DOI: 10.1107/S2414314624006539/bh4086Isup3.cml

CCDC reference: 2367491

Additional supporting information:  crystallographic information; 3D view; checkCIF report

## Figures and Tables

**Table 1:** Cremer & Pople analysis (Å, °)

Atom 1	Atom 2	Atom 3	Atom 4	Atom 5	Atom 6	*Q*	θ	φ	Conformation
C13*A*	C12*A*	C11*A*	C10*A*	N1*A*	O3*A*	0.581 (4)	5.7 (4)	14 (4)	Chair
O3	N1	C10	C11	C12	C13	0.581 (4)	5.9 (4)	350 (4)	Chair
C18	C19	C20	C21	C22	C23	0.808 (6)	52.7 (4)	243.4 (5)	Half-Boat
C18*A*	C19*A*	C20*A*	C21*A*	C22*A*	C23*A*	0.799 (8)	54.7 (6)	243.7 (7)	Half-Boat
C18*B*	C19*B*	C20*B*	C21*B*	C22*B*	C23*B*	0.78 (3)	54 (2)	258 (3)	Half-Boat
C18	C19	C20	C21	C23	C24	0.791 (6)	121.0 (4)	57.2 (5)	Half-Boat
C18*A*	C19*A*	C20*A*	C21*A*	C23*A*	C24*A*	0.829 (8)	122.5 (6)	56.2 (7)	Half-Boat
C18*B*	C19*B*	C20*B*	C21*B*	C23*B*	C24*B*	0.88 (3)	118 (2)	46 (2)	Half-Boat

**Table 2:** Experimental details

Crystal data	
Chemical formula	C_26_H_41_BrNO_4_^+^·Br^−^
*M*_r_	591.42
Crystal system, space group	Monoclinic, *P*2_1_
Temperature (K)	110
*a*, *b*, *c* (Å)	16.4966 (6), 8.8166 (3), 18.9475 (7)
β (°)	99.265 (2)
*V* (Å^3^)	2719.84 (17)
*Z*	4
Radiation type	Cu *K*α
μ (mm^−1^)	4.02
Crystal size (mm)	0.59 × 0.57 × 0.06

Data collection	
Diffractometer	Bruker D8 VENTURE dual wavelength Ag/Cu
Absorption correction	Multi-scan (*SADABS*; Krause *et al.*, 2015 ▸)
*T*_min_, *T*_max_	0.453, 0.598
No. of measured, independent and observed [*I* > 2σ(*I*)] reflections	103023, 9273, 9063
*R*_int_	0.044
(sin θ/λ)_max_ (Å^−1^)	0.595

Refinement	
*R*[*F*^2^ > 2σ(*F*^2^)], *wR*(*F*^2^), *S*	0.025, 0.063, 1.04
No. of reflections	9273
No. of parameters	650
No. of restraints	201
H-atom treatment	H-atom parameters constrained
Δρ_max_, Δρ_min_ (e Å^−3^)	0.68, −0.59
Absolute structure	Refined as an inversion twin.
Absolute structure parameter	−0.009 (17)

Computer programs: *APEX5* (Bruker, 2023 ▸), *SAINT* (Bruker, 2016 ▸), *SHELXT2018/2* (Sheldrick, 2015*a* ▸), *SHELXL2019/3* (Sheldrick, 2015*b* ▸), *ORTEP* (Burnett & Johnson, 1996 ▸), *Mercury* (Macrae *et al.*, 2020 ▸) and *OLEX2* (Dolomanov *et al.*, 2009 ▸).

## full crystallographic data

### 4-[(2-Bromo-4,5-dimethoxyphenyl)methyl]-4-{2-[2-(6,6-dimethyl-2-bicyclo[3.1.1]heptanyl)ethoxy]ethyl}morpholin-4-ium bromide Crystal data

**Table:**

C_26_H_41_BrNO_4_^+^·Br^−^	*F*(000) = 1224
*M**_r_* = 591.42	*D*_x_ = 1.444 Mg m^−^^3^
Monoclinic, *P*2_1_	Cu *K*α radiation, λ = 1.54178 Å
*a* = 16.4966 (6) Å	Cell parameters from 9547 reflections
*b* = 8.8166 (3) Å	θ = 3.3–66.5°
*c* = 18.9475 (7) Å	µ = 4.02 mm^−^^1^
β = 99.265 (2)°	*T* = 110 K
*V* = 2719.84 (17) Å^3^	Plate, clear light colourless
*Z* = 4	0.59 × 0.57 × 0.06 mm

### 4-[(2-Bromo-4,5-dimethoxyphenyl)methyl]-4-{2-[2-(6,6-dimethyl-2-bicyclo[3.1.1]heptanyl)ethoxy]ethyl}morpholin-4-ium bromide Data collection

**Table:**

Bruker D8 VENTURE dual wavelength Ag/Cu diffractometer	9273 independent reflections
Radiation source: microfocus sealed X-ray tube, Incoatec Iµs	9063 reflections with *I* > 2σ(*I*)
Mirror optics monochromator	*R*_int_ = 0.044
Detector resolution: 7.41 pixels mm^-1^	θ_max_ = 66.7°, θ_min_ = 2.4°
ω and φ scans	*h* = −19→19
Absorption correction: multi-scan (SADABS; Krause *et al.*, 2015)	*k* = −10→8
*T*_min_ = 0.453, *T*_max_ = 0.598	*l* = −22→22
103023 measured reflections	

### 4-[(2-Bromo-4,5-dimethoxyphenyl)methyl]-4-{2-[2-(6,6-dimethyl-2-bicyclo[3.1.1]heptanyl)ethoxy]ethyl}morpholin-4-ium bromide Refinement

**Table:**

Refinement on *F*^2^	Secondary atom site location: difference Fourier map
Least-squares matrix: full	Hydrogen site location: inferred from neighbouring sites
*R*[*F*^2^ > 2σ(*F*^2^)] = 0.025	H-atom parameters constrained
*wR*(*F*^2^) = 0.063	*w* = 1/[σ^2^(*F*_o_^2^) + (0.0278*P*)^2^ + 2.6359*P*] where *P* = (*F*_o_^2^ + 2*F*_c_^2^)/3
*S* = 1.04	(Δ/σ)_max_ = 0.002
9273 reflections	Δρ_max_ = 0.68 e Å^−^^3^
650 parameters	Δρ_min_ = −0.59 e Å^−^^3^
201 restraints	Absolute structure: Refined as an inversion twin.
Primary atom site location: dual	Absolute structure parameter: −0.009 (17)

### 4-[(2-Bromo-4,5-dimethoxyphenyl)methyl]-4-{2-[2-(6,6-dimethyl-2-bicyclo[3.1.1]heptanyl)ethoxy]ethyl}morpholin-4-ium bromide Special details

**Table:**

Refinement. Refined as a 2-component inversion twin.

### 4-[(2-Bromo-4,5-dimethoxyphenyl)methyl]-4-{2-[2-(6,6-dimethyl-2-bicyclo[3.1.1]heptanyl)ethoxy]ethyl}morpholin-4-ium bromide Fractional atomic coordinates and isotropic or equivalent isotropic displacement parameters (Å^2^)

**Table:**

	*x*	*y*	*z*	*U*_iso_*/*U*_eq_	Occ. (<1)
Br1	0.77782 (3)	0.65663 (5)	0.80156 (2)	0.02972 (11)	
O1	0.74510 (19)	0.1905 (4)	0.62731 (15)	0.0283 (7)	
O2	0.66700 (19)	0.0272 (4)	0.70686 (15)	0.0288 (7)	
O3	0.44575 (18)	0.6082 (4)	0.82426 (15)	0.0250 (7)	
O4	0.72470 (17)	0.5705 (4)	1.02623 (15)	0.0279 (7)	
N1	0.59756 (19)	0.4833 (4)	0.90094 (16)	0.0171 (7)	
C1	0.7961 (3)	0.2693 (6)	0.5848 (2)	0.0335 (11)	
H1A	0.808620	0.202202	0.546712	0.050*	
H1B	0.847313	0.300050	0.615027	0.050*	
H1C	0.767168	0.359478	0.563466	0.050*	
C2	0.6162 (3)	−0.0564 (6)	0.7470 (3)	0.0349 (12)	
H2A	0.601573	−0.153794	0.723396	0.052*	
H2B	0.566149	0.001499	0.749825	0.052*	
H2C	0.646011	−0.074463	0.795299	0.052*	
C3	0.6879 (2)	0.1684 (6)	0.7318 (2)	0.0207 (9)	
C4	0.6723 (2)	0.2263 (5)	0.7959 (2)	0.0189 (8)	
H4	0.645628	0.163709	0.825946	0.023*	
C5	0.6943 (2)	0.3743 (5)	0.8181 (2)	0.0184 (8)	
C6	0.7347 (2)	0.4613 (5)	0.7735 (2)	0.0204 (8)	
C7	0.7529 (2)	0.4053 (5)	0.7086 (2)	0.0218 (9)	
H7	0.780762	0.467205	0.679238	0.026*	
C8	0.7299 (3)	0.2595 (5)	0.6877 (2)	0.0222 (9)	
C9	0.6823 (2)	0.4173 (5)	0.8925 (2)	0.0203 (9)	
H9A	0.724835	0.492778	0.911109	0.024*	
H9B	0.692145	0.326036	0.923119	0.024*	
C10	0.5289 (2)	0.3872 (5)	0.8594 (2)	0.0204 (9)	
H10C	0.540206	0.370954	0.810148	0.025*	
H10D	0.528039	0.286717	0.882577	0.025*	
C11	0.4460 (2)	0.4618 (6)	0.8561 (2)	0.0234 (9)	
H11C	0.403371	0.397811	0.827750	0.028*	
H11D	0.432732	0.471320	0.904991	0.028*	
C12	0.5033 (3)	0.7033 (5)	0.8686 (2)	0.0244 (10)	
H12C	0.489436	0.706808	0.917556	0.029*	
H12D	0.500204	0.807746	0.849262	0.029*	
C13	0.5893 (2)	0.6419 (5)	0.8713 (2)	0.0195 (8)	
H13C	0.603396	0.642138	0.822458	0.023*	
H13D	0.628601	0.709209	0.901467	0.023*	
C14	0.5903 (2)	0.4773 (5)	0.98005 (19)	0.0186 (8)	
H14C	0.531791	0.491159	0.984373	0.022*	
H14D	0.606455	0.374376	0.997925	0.022*	
C15	0.6400 (2)	0.5906 (5)	1.0287 (2)	0.0216 (9)	
H15C	0.623215	0.694902	1.013327	0.026*	
H15D	0.629630	0.576578	1.078281	0.026*	
C16	0.7773 (3)	0.6458 (6)	1.0818 (2)	0.0322 (10)	
H16E	0.763432	0.617245	1.128959	0.039*	
H16F	0.771695	0.757160	1.076117	0.039*	
C17	0.8632 (3)	0.5968 (6)	1.0760 (2)	0.0349 (11)	
H17E	0.876736	0.633690	1.029984	0.042*	
H17F	0.864931	0.484608	1.075165	0.042*	
C18	0.9294 (3)	0.6538 (7)	1.1369 (2)	0.0384 (12)	
H18	0.908993	0.628652	1.182447	0.046*	
C19	0.9405 (3)	0.8255 (6)	1.1371 (3)	0.0426 (12)	
H19E	0.913238	0.869173	1.175253	0.051*	
H19F	0.912364	0.866094	1.090844	0.051*	
C20	1.0328 (3)	0.8805 (7)	1.1489 (4)	0.0571 (16)	
H20E	1.041115	0.948398	1.109000	0.068*	
H20F	1.044019	0.939879	1.193723	0.068*	
C21	1.0926 (3)	0.7515 (7)	1.1529 (3)	0.0475 (14)	
H21	1.151664	0.782512	1.161165	0.057*	
C22	1.0704 (3)	0.6311 (9)	1.2044 (3)	0.0619 (18)	
H22E	1.043354	0.671396	1.243475	0.074*	
H22F	1.115941	0.561354	1.222861	0.074*	
C23	1.0108 (3)	0.5695 (7)	1.1399 (3)	0.0454 (14)	
H23	1.005797	0.456469	1.138258	0.054*	
C24	1.0693 (3)	0.6361 (7)	1.0920 (3)	0.0409 (12)	
C25	1.0364 (3)	0.6954 (8)	1.0169 (3)	0.0534 (16)	
H25G	0.992427	0.768894	1.019516	0.080*	
H25H	1.014769	0.610525	0.986114	0.080*	
H25I	1.080966	0.744657	0.997022	0.080*	
C26	1.1404 (3)	0.5297 (8)	1.0837 (4)	0.0587 (16)	
H26G	1.184495	0.587729	1.067268	0.088*	
H26H	1.121058	0.450640	1.048721	0.088*	
H26I	1.161296	0.482723	1.129962	0.088*	
Br1A	0.20805 (3)	0.34247 (6)	0.67939 (3)	0.03860 (13)	
O1A	0.2564 (2)	0.7808 (4)	0.86903 (16)	0.0317 (7)	
O2A	0.3262 (2)	0.9594 (4)	0.78952 (16)	0.0301 (7)	
O3A	0.54323 (18)	0.3963 (4)	0.67381 (15)	0.0250 (7)	
O4A	0.28184 (19)	0.3885 (4)	0.46950 (17)	0.0332 (8)	
N1A	0.39434 (19)	0.5208 (4)	0.59104 (16)	0.0168 (7)	
C1A	0.2193 (3)	0.6863 (6)	0.9161 (2)	0.0327 (11)	
H1AA	0.164551	0.655166	0.892476	0.049*	
H1AB	0.214338	0.742724	0.959817	0.049*	
H1AC	0.253391	0.596183	0.928441	0.049*	
C2A	0.3719 (3)	1.0521 (6)	0.7487 (3)	0.0378 (12)	
H2AA	0.338294	1.075053	0.702386	0.057*	
H2AB	0.421698	0.998337	0.740967	0.057*	
H2AC	0.387073	1.146838	0.774501	0.057*	
C3A	0.3023 (3)	0.8204 (5)	0.7606 (2)	0.0231 (9)	
C4A	0.3145 (3)	0.7728 (5)	0.6944 (2)	0.0229 (9)	
H4A	0.339054	0.840387	0.665023	0.027*	
C5A	0.2918 (2)	0.6273 (5)	0.6685 (2)	0.0233 (9)	
C6A	0.2519 (2)	0.5357 (6)	0.7115 (2)	0.0255 (9)	
C7A	0.2391 (3)	0.5820 (6)	0.7789 (2)	0.0278 (10)	
H7A	0.212318	0.515796	0.807339	0.033*	
C8A	0.2647 (3)	0.7230 (6)	0.8049 (2)	0.0250 (10)	
C9A	0.3077 (2)	0.5867 (5)	0.5951 (2)	0.0222 (9)	
H9AA	0.299721	0.678698	0.564866	0.027*	
H9AB	0.266041	0.511471	0.574369	0.027*	
C10A	0.4009 (2)	0.3618 (5)	0.6213 (2)	0.0193 (9)	
H10A	0.363417	0.293911	0.589713	0.023*	
H10B	0.383753	0.361891	0.668997	0.023*	
C11A	0.4883 (3)	0.3021 (5)	0.6279 (2)	0.0219 (9)	
H11A	0.490717	0.197534	0.647167	0.026*	
H11B	0.504970	0.298821	0.579993	0.026*	
C12A	0.5445 (2)	0.5425 (6)	0.6422 (2)	0.0217 (9)	
H12A	0.560911	0.533013	0.594394	0.026*	
H12B	0.585590	0.606898	0.672165	0.026*	
C13A	0.4607 (2)	0.6168 (5)	0.6350 (2)	0.0195 (9)	
H13A	0.462790	0.717154	0.612007	0.023*	
H13B	0.446365	0.633043	0.683244	0.023*	
C14A	0.4057 (2)	0.5283 (6)	0.51247 (19)	0.0194 (8)	
H14A	0.382413	0.625276	0.492064	0.023*	
H14B	0.465316	0.529671	0.510442	0.023*	
C15A	0.3667 (3)	0.3994 (6)	0.4656 (2)	0.0245 (9)	
H15A	0.374012	0.417507	0.415461	0.029*	
H15B	0.394270	0.302779	0.481577	0.029*	
C16A	0.2479 (4)	0.2667 (9)	0.4199 (4)	0.0395 (16)	0.78
H16A	0.288671	0.184356	0.419452	0.047*	0.78
H16B	0.232894	0.306725	0.370749	0.047*	0.78
C17A	0.1700 (4)	0.2064 (8)	0.4485 (4)	0.0435 (14)	0.78
H17A	0.152482	0.110209	0.423699	0.052*	0.78
H17B	0.185087	0.183721	0.500083	0.052*	0.78
C18A	0.0982 (4)	0.3153 (7)	0.4383 (3)	0.0348 (12)	0.78
H18A	0.120076	0.413840	0.459590	0.042*	0.78
C19A	0.0307 (5)	0.2617 (12)	0.4847 (4)	0.063 (2)	0.78
H19A	0.043444	0.156553	0.501182	0.076*	0.78
H19B	0.034752	0.326968	0.527660	0.076*	0.78
C20A	−0.0580 (5)	0.2669 (12)	0.4448 (5)	0.067 (2)	0.78
H20A	−0.082280	0.164278	0.444861	0.080*	0.78
H20B	−0.090621	0.335570	0.470587	0.080*	0.78
C21A	−0.0638 (4)	0.3207 (11)	0.3685 (4)	0.0503 (14)	0.78
H21A	−0.121356	0.328332	0.342351	0.060*	0.78
C22A	−0.0138 (4)	0.4600 (11)	0.3639 (4)	0.0539 (15)	0.78
H22A	−0.008902	0.526198	0.406570	0.065*	0.78
H22B	−0.029471	0.518761	0.319239	0.065*	0.78
C23A	0.0607 (4)	0.3499 (9)	0.3630 (3)	0.0405 (12)	0.78
H23A	0.100648	0.382835	0.331541	0.049*	0.78
C24A	−0.0053 (4)	0.2275 (9)	0.3281 (4)	0.0437 (13)	0.78
C25A	−0.0245 (5)	0.2498 (11)	0.2482 (4)	0.0559 (18)	0.78
H25A	−0.072674	0.189211	0.228723	0.084*	0.78
H25B	0.022685	0.217538	0.226414	0.084*	0.78
H25C	−0.035816	0.357285	0.237664	0.084*	0.78
C26A	0.0064 (5)	0.0599 (9)	0.3450 (4)	0.0560 (17)	0.78
H26A	0.012921	0.044855	0.396831	0.084*	0.78
H26B	0.055533	0.023309	0.327355	0.084*	0.78
H26C	−0.041732	0.003456	0.321663	0.084*	0.78
C16B	0.2245 (15)	0.300 (4)	0.4340 (17)	0.047 (8)*	0.22
H16C	0.237372	0.193649	0.447749	0.056*	0.22
H16D	0.226331	0.310007	0.382264	0.056*	0.22
C17B	0.1336 (14)	0.337 (4)	0.4474 (14)	0.051 (8)*	0.22
H17C	0.132930	0.336088	0.499522	0.061*	0.22
H17D	0.119077	0.440997	0.429898	0.061*	0.22
C18B	0.0692 (13)	0.230 (4)	0.4122 (13)	0.069 (7)*	0.22
H18B	0.083964	0.123717	0.427243	0.083*	0.22
C19B	0.0657 (13)	0.248 (4)	0.3264 (11)	0.057 (7)*	0.22
H19C	0.098621	0.336974	0.316512	0.068*	0.22
H19D	0.090038	0.156703	0.307524	0.068*	0.22
C20B	−0.0262 (13)	0.268 (3)	0.2871 (12)	0.040 (5)*	0.22
H20C	−0.030741	0.366758	0.261903	0.048*	0.22
H20D	−0.038246	0.187548	0.250470	0.048*	0.22
C21B	−0.0891 (15)	0.262 (3)	0.3342 (13)	0.061 (8)*	0.22
H21B	−0.146926	0.265007	0.308460	0.074*	0.22
C22B	−0.0755 (16)	0.156 (3)	0.3963 (14)	0.067 (7)*	0.22
H22C	−0.051034	0.057267	0.386094	0.080*	0.22
H22D	−0.124015	0.141715	0.420312	0.080*	0.22
C23B	−0.0134 (13)	0.272 (3)	0.4325 (12)	0.054 (6)*	0.22
H23B	−0.012584	0.281741	0.485172	0.065*	0.22
C24B	−0.0655 (14)	0.397 (3)	0.3872 (13)	0.047 (6)*	0.22
C25B	−0.1405 (18)	0.437 (4)	0.4222 (18)	0.083 (11)*	0.22
H25D	−0.167598	0.526745	0.398976	0.124*	0.22
H25E	−0.122643	0.457443	0.473113	0.124*	0.22
H25F	−0.178984	0.351312	0.416727	0.124*	0.22
C26B	−0.036 (3)	0.550 (4)	0.365 (2)	0.104 (13)*	0.22
H26D	−0.009296	0.536913	0.322277	0.155*	0.22
H26E	0.003778	0.592590	0.403711	0.155*	0.22
H26F	−0.082647	0.618935	0.353278	0.155*	0.22
Br2	0.60599 (3)	1.02818 (5)	0.95233 (2)	0.02366 (10)	
Br2A	0.38707 (3)	−0.02718 (5)	0.54091 (2)	0.02466 (11)	

### 4-[(2-Bromo-4,5-dimethoxyphenyl)methyl]-4-{2-[2-(6,6-dimethyl-2-bicyclo[3.1.1]heptanyl)ethoxy]ethyl}morpholin-4-ium bromide Atomic displacement parameters (Å^2^)

**Table:**

	*U*^11^	*U*^22^	*U*^33^	*U*^12^	*U*^13^	*U*^23^
Br1	0.0312 (2)	0.0221 (3)	0.0393 (2)	−0.00864 (19)	0.01631 (19)	−0.0048 (2)
O1	0.0358 (16)	0.032 (2)	0.0198 (14)	−0.0079 (14)	0.0136 (12)	−0.0044 (12)
O2	0.0429 (18)	0.0205 (17)	0.0254 (14)	−0.0068 (15)	0.0125 (13)	−0.0061 (14)
O3	0.0252 (15)	0.0256 (18)	0.0232 (15)	0.0038 (13)	0.0006 (12)	0.0002 (12)
O4	0.0194 (14)	0.039 (2)	0.0243 (15)	−0.0073 (13)	0.0005 (11)	−0.0069 (13)
N1	0.0167 (15)	0.0177 (19)	0.0173 (15)	−0.0001 (14)	0.0039 (12)	−0.0015 (14)
C1	0.035 (3)	0.043 (3)	0.026 (2)	−0.006 (2)	0.0144 (19)	−0.002 (2)
C2	0.048 (3)	0.025 (3)	0.035 (2)	−0.017 (2)	0.018 (2)	−0.0041 (19)
C3	0.0207 (19)	0.022 (2)	0.0190 (19)	−0.0028 (18)	0.0036 (15)	−0.0002 (17)
C4	0.0190 (19)	0.019 (2)	0.0195 (18)	−0.0006 (16)	0.0046 (15)	0.0034 (16)
C5	0.0144 (18)	0.020 (2)	0.0211 (19)	0.0009 (15)	0.0036 (14)	0.0005 (15)
C6	0.0184 (19)	0.016 (2)	0.0269 (19)	−0.0004 (17)	0.0029 (15)	−0.0013 (17)
C7	0.019 (2)	0.025 (2)	0.023 (2)	−0.0016 (16)	0.0081 (16)	0.0032 (17)
C8	0.021 (2)	0.026 (3)	0.020 (2)	−0.0016 (18)	0.0030 (16)	−0.0011 (16)
C9	0.0187 (19)	0.019 (2)	0.023 (2)	0.0005 (16)	0.0032 (16)	−0.0004 (16)
C10	0.020 (2)	0.022 (3)	0.0191 (19)	−0.0037 (17)	0.0037 (16)	−0.0037 (16)
C11	0.0194 (19)	0.024 (2)	0.026 (2)	−0.0014 (18)	0.0008 (16)	−0.0027 (18)
C12	0.028 (2)	0.020 (3)	0.025 (2)	0.0017 (18)	0.0055 (18)	−0.0014 (17)
C13	0.026 (2)	0.014 (2)	0.0193 (19)	−0.0025 (17)	0.0057 (15)	0.0015 (16)
C14	0.0197 (19)	0.020 (2)	0.0167 (17)	−0.0018 (17)	0.0047 (14)	0.0025 (17)
C15	0.023 (2)	0.026 (2)	0.0168 (19)	0.0001 (17)	0.0053 (15)	−0.0015 (16)
C16	0.032 (2)	0.037 (3)	0.027 (2)	−0.011 (2)	0.0026 (17)	−0.008 (2)
C17	0.028 (2)	0.041 (3)	0.035 (2)	−0.009 (2)	0.0030 (19)	−0.006 (2)
C18	0.035 (2)	0.048 (3)	0.032 (2)	−0.012 (2)	0.005 (2)	0.003 (2)
C19	0.029 (2)	0.046 (3)	0.054 (3)	−0.009 (2)	0.008 (2)	−0.019 (3)
C20	0.035 (3)	0.046 (4)	0.089 (4)	−0.013 (2)	0.007 (3)	−0.024 (3)
C21	0.024 (2)	0.055 (4)	0.062 (3)	−0.017 (2)	0.002 (2)	−0.003 (3)
C22	0.037 (3)	0.096 (5)	0.049 (3)	−0.013 (3)	−0.003 (2)	0.014 (3)
C23	0.033 (3)	0.048 (4)	0.052 (3)	−0.008 (2)	−0.001 (2)	0.012 (3)
C24	0.022 (2)	0.046 (3)	0.054 (3)	−0.003 (2)	0.004 (2)	−0.006 (3)
C25	0.037 (3)	0.077 (5)	0.051 (3)	0.006 (3)	0.019 (2)	−0.002 (3)
C26	0.035 (3)	0.057 (4)	0.083 (4)	0.004 (3)	0.006 (3)	−0.008 (3)
Br1A	0.0351 (3)	0.0238 (3)	0.0630 (3)	−0.0082 (2)	0.0265 (2)	−0.0053 (2)
O1A	0.0395 (18)	0.0315 (19)	0.0281 (16)	0.0004 (14)	0.0173 (14)	0.0038 (13)
O2A	0.0416 (18)	0.0226 (18)	0.0306 (15)	−0.0035 (15)	0.0190 (13)	−0.0010 (14)
O3A	0.0243 (15)	0.0265 (18)	0.0226 (14)	0.0028 (13)	−0.0010 (12)	0.0001 (12)
O4A	0.0256 (16)	0.037 (2)	0.0340 (17)	−0.0047 (14)	−0.0044 (13)	−0.0026 (14)
N1A	0.0172 (15)	0.0150 (19)	0.0194 (15)	−0.0013 (14)	0.0064 (12)	0.0003 (14)
C1A	0.033 (2)	0.043 (3)	0.025 (2)	0.000 (2)	0.0131 (18)	0.008 (2)
C2A	0.056 (3)	0.023 (3)	0.039 (3)	−0.010 (2)	0.021 (2)	−0.006 (2)
C3A	0.026 (2)	0.012 (2)	0.033 (2)	0.0051 (17)	0.0123 (17)	0.0031 (17)
C4A	0.024 (2)	0.018 (2)	0.030 (2)	0.0049 (17)	0.0158 (17)	0.0046 (17)
C5A	0.023 (2)	0.018 (3)	0.032 (2)	0.0028 (17)	0.0116 (17)	0.0015 (17)
C6A	0.023 (2)	0.019 (2)	0.038 (2)	−0.0006 (18)	0.0145 (17)	0.0010 (19)
C7A	0.024 (2)	0.027 (3)	0.037 (2)	0.0032 (17)	0.0165 (19)	0.0075 (19)
C8A	0.024 (2)	0.028 (3)	0.026 (2)	0.0075 (19)	0.0151 (17)	0.0056 (18)
C9A	0.0176 (19)	0.020 (2)	0.031 (2)	0.0031 (16)	0.0111 (17)	0.0005 (17)
C10A	0.023 (2)	0.016 (2)	0.0200 (19)	0.0002 (17)	0.0066 (15)	0.0015 (16)
C11A	0.026 (2)	0.017 (2)	0.023 (2)	0.0030 (17)	0.0055 (17)	0.0007 (16)
C12A	0.022 (2)	0.021 (2)	0.0218 (19)	−0.0022 (18)	0.0025 (15)	−0.0031 (17)
C13A	0.022 (2)	0.016 (2)	0.0203 (19)	−0.0031 (16)	0.0023 (16)	−0.0055 (15)
C14A	0.0199 (19)	0.023 (2)	0.0159 (17)	0.0021 (18)	0.0059 (14)	0.0018 (17)
C15A	0.024 (2)	0.028 (3)	0.0191 (19)	0.0007 (18)	−0.0036 (16)	−0.0005 (17)
C16A	0.028 (3)	0.040 (4)	0.048 (4)	−0.004 (3)	−0.001 (3)	−0.029 (3)
C17A	0.038 (3)	0.038 (4)	0.050 (4)	−0.005 (2)	−0.003 (2)	−0.005 (3)
C18A	0.037 (3)	0.028 (3)	0.040 (3)	−0.007 (2)	0.009 (2)	−0.003 (2)
C19A	0.060 (3)	0.090 (6)	0.043 (3)	−0.011 (4)	0.020 (2)	0.003 (4)
C20A	0.046 (3)	0.093 (6)	0.070 (4)	0.013 (4)	0.034 (3)	0.026 (4)
C21A	0.032 (3)	0.065 (4)	0.057 (3)	0.005 (3)	0.017 (3)	0.007 (3)
C22A	0.042 (3)	0.059 (3)	0.064 (4)	0.007 (3)	0.018 (3)	0.006 (3)
C23A	0.034 (2)	0.045 (3)	0.045 (2)	−0.001 (2)	0.013 (2)	0.007 (3)
C24A	0.029 (3)	0.054 (3)	0.050 (3)	−0.004 (2)	0.011 (2)	0.005 (2)
C25A	0.046 (4)	0.072 (5)	0.050 (3)	0.002 (4)	0.008 (3)	−0.003 (3)
C26A	0.045 (4)	0.051 (3)	0.068 (5)	−0.012 (3)	−0.003 (3)	−0.001 (3)
Br2	0.0300 (2)	0.0190 (2)	0.0241 (2)	−0.00420 (18)	0.01098 (17)	0.00046 (16)
Br2A	0.0318 (2)	0.0181 (2)	0.0269 (2)	−0.00198 (18)	0.01295 (17)	0.00165 (17)

### 4-[(2-Bromo-4,5-dimethoxyphenyl)methyl]-4-{2-[2-(6,6-dimethyl-2-bicyclo[3.1.1]heptanyl)ethoxy]ethyl}morpholin-4-ium bromide Geometric parameters (Å, º)

**Table:**

Br1—C6	1.906 (5)	C1A—H1AC	0.9800
O1—C1	1.435 (5)	C2A—H2AA	0.9800
O1—C8	1.354 (5)	C2A—H2AB	0.9800
O2—C2	1.424 (6)	C2A—H2AC	0.9800
O2—C3	1.356 (6)	C3A—C4A	1.367 (6)
O3—C11	1.424 (6)	C3A—C8A	1.413 (6)
O3—C12	1.433 (5)	C4A—H4A	0.9500
O4—C15	1.417 (5)	C4A—C5A	1.402 (6)
O4—C16	1.417 (5)	C5A—C6A	1.387 (6)
N1—C9	1.546 (5)	C5A—C9A	1.499 (6)
N1—C10	1.528 (5)	C6A—C7A	1.388 (6)
N1—C13	1.505 (6)	C7A—H7A	0.9500
N1—C14	1.524 (4)	C7A—C8A	1.379 (7)
C1—H1A	0.9800	C9A—H9AA	0.9900
C1—H1B	0.9800	C9A—H9AB	0.9900
C1—H1C	0.9800	C10A—H10A	0.9900
C2—H2A	0.9800	C10A—H10B	0.9900
C2—H2B	0.9800	C10A—C11A	1.520 (6)
C2—H2C	0.9800	C11A—H11A	0.9900
C3—C4	1.380 (6)	C11A—H11B	0.9900
C3—C8	1.417 (6)	C12A—H12A	0.9900
C4—H4	0.9500	C12A—H12B	0.9900
C4—C5	1.401 (6)	C12A—C13A	1.517 (6)
C5—C6	1.388 (6)	C13A—H13A	0.9900
C5—C9	1.503 (5)	C13A—H13B	0.9900
C6—C7	1.402 (6)	C14A—H14A	0.9900
C7—H7	0.9500	C14A—H14B	0.9900
C7—C8	1.380 (7)	C14A—C15A	1.520 (6)
C9—H9A	0.9900	C15A—H15A	0.9900
C9—H9B	0.9900	C15A—H15B	0.9900
C10—H10C	0.9900	C16A—H16A	0.9900
C10—H10D	0.9900	C16A—H16B	0.9900
C10—C11	1.510 (6)	C16A—C17A	1.565 (10)
C11—H11C	0.9900	C17A—H17A	0.9900
C11—H11D	0.9900	C17A—H17B	0.9900
C12—H12C	0.9900	C17A—C18A	1.514 (9)
C12—H12D	0.9900	C18A—H18A	1.0000
C12—C13	1.511 (6)	C18A—C19A	1.596 (9)
C13—H13C	0.9900	C18A—C23A	1.492 (9)
C13—H13D	0.9900	C19A—H19A	0.9900
C14—H14C	0.9900	C19A—H19B	0.9900
C14—H14D	0.9900	C19A—C20A	1.536 (11)
C14—C15	1.508 (6)	C20A—H20A	0.9900
C15—H15C	0.9900	C20A—H20B	0.9900
C15—H15D	0.9900	C20A—C21A	1.509 (11)
C16—H16E	0.9900	C21A—H21A	1.0000
C16—H16F	0.9900	C21A—C22A	1.490 (12)
C16—C17	1.502 (6)	C21A—C24A	1.559 (10)
C17—H17E	0.9900	C22A—H22A	0.9900
C17—H17F	0.9900	C22A—H22B	0.9900
C17—C18	1.541 (6)	C22A—C23A	1.569 (10)
C18—H18	1.0000	C23A—H23A	1.0000
C18—C19	1.524 (8)	C23A—C24A	1.599 (10)
C18—C23	1.527 (8)	C24A—C25A	1.508 (10)
C19—H19E	0.9900	C24A—C26A	1.518 (11)
C19—H19F	0.9900	C25A—H25A	0.9800
C19—C20	1.579 (7)	C25A—H25B	0.9800
C20—H20E	0.9900	C25A—H25C	0.9800
C20—H20F	0.9900	C26A—H26A	0.9800
C20—C21	1.499 (8)	C26A—H26B	0.9800
C21—H21	1.0000	C26A—H26C	0.9800
C21—C22	1.526 (9)	C16B—H16C	0.9900
C21—C24	1.541 (8)	C16B—H16D	0.9900
C22—H22E	0.9900	C16B—C17B	1.59 (2)
C22—H22F	0.9900	C17B—H17C	0.9900
C22—C23	1.539 (7)	C17B—H17D	0.9900
C23—H23	1.0000	C17B—C18B	1.49 (2)
C23—C24	1.544 (7)	C18B—H18B	1.0000
C24—C25	1.530 (8)	C18B—C19B	1.62 (2)
C24—C26	1.530 (8)	C18B—C23B	1.52 (2)
C25—H25G	0.9800	C19B—H19C	0.9900
C25—H25H	0.9800	C19B—H19D	0.9900
C25—H25I	0.9800	C19B—C20B	1.59 (2)
C26—H26G	0.9800	C20B—H20C	0.9900
C26—H26H	0.9800	C20B—H20D	0.9900
C26—H26I	0.9800	C20B—C21B	1.47 (2)
Br1A—C6A	1.912 (5)	C21B—H21B	1.0000
O1A—C1A	1.430 (5)	C21B—C22B	1.49 (3)
O1A—C8A	1.345 (5)	C21B—C24B	1.56 (2)
O2A—C2A	1.422 (6)	C22B—H22C	0.9900
O2A—C3A	1.375 (6)	C22B—H22D	0.9900
O3A—C11A	1.420 (5)	C22B—C23B	1.53 (2)
O3A—C12A	1.422 (6)	C23B—H23B	1.0000
O4A—C15A	1.417 (5)	C23B—C24B	1.57 (2)
O4A—C16A	1.477 (7)	C24B—C25B	1.53 (2)
O4A—C16B	1.32 (3)	C24B—C26B	1.52 (2)
N1A—C9A	1.556 (5)	C25B—H25D	0.9800
N1A—C10A	1.512 (5)	C25B—H25E	0.9800
N1A—C13A	1.521 (5)	C25B—H25F	0.9800
N1A—C14A	1.532 (4)	C26B—H26D	0.9800
C1A—H1AA	0.9800	C26B—H26E	0.9800
C1A—H1AB	0.9800	C26B—H26F	0.9800
			
C8—O1—C1	117.6 (4)	C7A—C6A—Br1A	116.7 (3)
C3—O2—C2	115.4 (3)	C6A—C7A—H7A	119.7
C11—O3—C12	109.0 (3)	C8A—C7A—C6A	120.7 (4)
C16—O4—C15	114.0 (3)	C8A—C7A—H7A	119.7
C10—N1—C9	110.2 (3)	O1A—C8A—C3A	115.2 (4)
C13—N1—C9	109.9 (3)	O1A—C8A—C7A	126.5 (4)
C13—N1—C10	107.9 (3)	C7A—C8A—C3A	118.3 (4)
C13—N1—C14	112.5 (3)	N1A—C9A—H9AA	108.3
C14—N1—C9	107.7 (3)	N1A—C9A—H9AB	108.3
C14—N1—C10	108.6 (3)	C5A—C9A—N1A	115.8 (3)
O1—C1—H1A	109.5	C5A—C9A—H9AA	108.3
O1—C1—H1B	109.5	C5A—C9A—H9AB	108.3
O1—C1—H1C	109.5	H9AA—C9A—H9AB	107.4
H1A—C1—H1B	109.5	N1A—C10A—H10A	109.4
H1A—C1—H1C	109.5	N1A—C10A—H10B	109.4
H1B—C1—H1C	109.5	N1A—C10A—C11A	111.2 (3)
O2—C2—H2A	109.5	H10A—C10A—H10B	108.0
O2—C2—H2B	109.5	C11A—C10A—H10A	109.4
O2—C2—H2C	109.5	C11A—C10A—H10B	109.4
H2A—C2—H2B	109.5	O3A—C11A—C10A	110.5 (4)
H2A—C2—H2C	109.5	O3A—C11A—H11A	109.5
H2B—C2—H2C	109.5	O3A—C11A—H11B	109.5
O2—C3—C4	124.9 (4)	C10A—C11A—H11A	109.5
O2—C3—C8	115.9 (4)	C10A—C11A—H11B	109.5
C4—C3—C8	119.2 (4)	H11A—C11A—H11B	108.1
C3—C4—H4	118.9	O3A—C12A—H12A	109.5
C3—C4—C5	122.2 (4)	O3A—C12A—H12B	109.5
C5—C4—H4	118.9	O3A—C12A—C13A	110.7 (3)
C4—C5—C9	116.7 (4)	H12A—C12A—H12B	108.1
C6—C5—C4	117.3 (4)	C13A—C12A—H12A	109.5
C6—C5—C9	125.4 (4)	C13A—C12A—H12B	109.5
C5—C6—Br1	121.6 (3)	N1A—C13A—H13A	109.2
C5—C6—C7	122.0 (4)	N1A—C13A—H13B	109.2
C7—C6—Br1	116.1 (3)	C12A—C13A—N1A	112.0 (3)
C6—C7—H7	120.3	C12A—C13A—H13A	109.2
C8—C7—C6	119.5 (4)	C12A—C13A—H13B	109.2
C8—C7—H7	120.3	H13A—C13A—H13B	107.9
O1—C8—C3	114.7 (4)	N1A—C14A—H14A	108.4
O1—C8—C7	125.5 (4)	N1A—C14A—H14B	108.4
C7—C8—C3	119.8 (4)	H14A—C14A—H14B	107.5
N1—C9—H9A	108.1	C15A—C14A—N1A	115.4 (4)
N1—C9—H9B	108.1	C15A—C14A—H14A	108.4
C5—C9—N1	116.7 (3)	C15A—C14A—H14B	108.4
C5—C9—H9A	108.1	O4A—C15A—C14A	110.5 (4)
C5—C9—H9B	108.1	O4A—C15A—H15A	109.6
H9A—C9—H9B	107.3	O4A—C15A—H15B	109.6
N1—C10—H10C	109.3	C14A—C15A—H15A	109.6
N1—C10—H10D	109.3	C14A—C15A—H15B	109.6
H10C—C10—H10D	107.9	H15A—C15A—H15B	108.1
C11—C10—N1	111.7 (4)	O4A—C16A—H16A	110.5
C11—C10—H10C	109.3	O4A—C16A—H16B	110.5
C11—C10—H10D	109.3	O4A—C16A—C17A	106.0 (5)
O3—C11—C10	110.7 (3)	H16A—C16A—H16B	108.7
O3—C11—H11C	109.5	C17A—C16A—H16A	110.5
O3—C11—H11D	109.5	C17A—C16A—H16B	110.5
C10—C11—H11C	109.5	C16A—C17A—H17A	108.7
C10—C11—H11D	109.5	C16A—C17A—H17B	108.7
H11C—C11—H11D	108.1	H17A—C17A—H17B	107.6
O3—C12—H12C	109.7	C18A—C17A—C16A	114.3 (6)
O3—C12—H12D	109.7	C18A—C17A—H17A	108.7
O3—C12—C13	109.9 (4)	C18A—C17A—H17B	108.7
H12C—C12—H12D	108.2	C17A—C18A—H18A	105.9
C13—C12—H12C	109.7	C17A—C18A—C19A	110.1 (6)
C13—C12—H12D	109.7	C19A—C18A—H18A	105.9
N1—C13—C12	111.9 (3)	C23A—C18A—C17A	116.5 (6)
N1—C13—H13C	109.2	C23A—C18A—H18A	105.9
N1—C13—H13D	109.2	C23A—C18A—C19A	111.7 (6)
C12—C13—H13C	109.2	C18A—C19A—H19A	108.7
C12—C13—H13D	109.2	C18A—C19A—H19B	108.7
H13C—C13—H13D	107.9	H19A—C19A—H19B	107.6
N1—C14—H14C	108.0	C20A—C19A—C18A	114.4 (6)
N1—C14—H14D	108.0	C20A—C19A—H19A	108.7
H14C—C14—H14D	107.2	C20A—C19A—H19B	108.7
C15—C14—N1	117.2 (4)	C19A—C20A—H20A	109.0
C15—C14—H14C	108.0	C19A—C20A—H20B	109.0
C15—C14—H14D	108.0	H20A—C20A—H20B	107.8
O4—C15—C14	109.8 (3)	C21A—C20A—C19A	112.9 (6)
O4—C15—H15C	109.7	C21A—C20A—H20A	109.0
O4—C15—H15D	109.7	C21A—C20A—H20B	109.0
C14—C15—H15C	109.7	C20A—C21A—H21A	113.9
C14—C15—H15D	109.7	C20A—C21A—C24A	110.9 (7)
H15C—C15—H15D	108.2	C22A—C21A—C20A	111.4 (8)
O4—C16—H16E	110.4	C22A—C21A—H21A	113.9
O4—C16—H16F	110.4	C22A—C21A—C24A	90.8 (5)
O4—C16—C17	106.4 (4)	C24A—C21A—H21A	113.9
H16E—C16—H16F	108.6	C21A—C22A—H22A	114.3
C17—C16—H16E	110.4	C21A—C22A—H22B	114.3
C17—C16—H16F	110.4	C21A—C22A—C23A	86.1 (6)
C16—C17—H17E	108.6	H22A—C22A—H22B	111.4
C16—C17—H17F	108.6	C23A—C22A—H22A	114.3
C16—C17—C18	114.6 (4)	C23A—C22A—H22B	114.3
H17E—C17—H17F	107.6	C18A—C23A—C22A	108.7 (6)
C18—C17—H17E	108.6	C18A—C23A—H23A	114.8
C18—C17—H17F	108.6	C18A—C23A—C24A	114.0 (6)
C17—C18—H18	106.0	C22A—C23A—H23A	114.8
C19—C18—C17	113.3 (5)	C22A—C23A—C24A	86.5 (5)
C19—C18—H18	106.0	C24A—C23A—H23A	114.8
C19—C18—C23	112.2 (4)	C21A—C24A—C23A	82.9 (6)
C23—C18—C17	112.5 (5)	C25A—C24A—C21A	112.2 (6)
C23—C18—H18	106.0	C25A—C24A—C23A	110.2 (6)
C18—C19—H19E	108.6	C25A—C24A—C26A	109.7 (7)
C18—C19—H19F	108.6	C26A—C24A—C21A	118.5 (7)
C18—C19—C20	114.7 (5)	C26A—C24A—C23A	121.0 (6)
H19E—C19—H19F	107.6	C24A—C25A—H25A	109.5
C20—C19—H19E	108.6	C24A—C25A—H25B	109.5
C20—C19—H19F	108.6	C24A—C25A—H25C	109.5
C19—C20—H20E	109.1	H25A—C25A—H25B	109.5
C19—C20—H20F	109.1	H25A—C25A—H25C	109.5
H20E—C20—H20F	107.8	H25B—C25A—H25C	109.5
C21—C20—C19	112.6 (5)	C24A—C26A—H26A	109.5
C21—C20—H20E	109.1	C24A—C26A—H26B	109.5
C21—C20—H20F	109.1	C24A—C26A—H26C	109.5
C20—C21—H21	114.7	H26A—C26A—H26B	109.5
C20—C21—C22	109.7 (5)	H26A—C26A—H26C	109.5
C20—C21—C24	112.3 (4)	H26B—C26A—H26C	109.5
C22—C21—H21	114.7	O4A—C16B—H16C	108.6
C22—C21—C24	87.9 (5)	O4A—C16B—H16D	108.6
C24—C21—H21	114.7	O4A—C16B—C17B	115 (2)
C21—C22—H22E	114.4	H16C—C16B—H16D	107.6
C21—C22—H22F	114.4	C17B—C16B—H16C	108.6
C21—C22—C23	85.8 (4)	C17B—C16B—H16D	108.6
H22E—C22—H22F	111.5	C16B—C17B—H17C	108.6
C23—C22—H22E	114.4	C16B—C17B—H17D	108.6
C23—C22—H22F	114.4	H17C—C17B—H17D	107.5
C18—C23—C22	107.7 (5)	C18B—C17B—C16B	115 (2)
C18—C23—H23	114.6	C18B—C17B—H17C	108.6
C18—C23—C24	114.9 (5)	C18B—C17B—H17D	108.6
C22—C23—H23	114.6	C17B—C18B—H18B	110.0
C22—C23—C24	87.3 (4)	C17B—C18B—C19B	107 (2)
C24—C23—H23	114.6	C17B—C18B—C23B	110 (2)
C21—C24—C23	85.1 (4)	C19B—C18B—H18B	110.0
C25—C24—C21	118.6 (5)	C23B—C18B—H18B	110.0
C25—C24—C23	121.1 (4)	C23B—C18B—C19B	109.9 (18)
C25—C24—C26	106.1 (5)	C18B—C19B—H19C	109.5
C26—C24—C21	112.2 (4)	C18B—C19B—H19D	109.5
C26—C24—C23	112.9 (5)	H19C—C19B—H19D	108.0
C24—C25—H25G	109.5	C20B—C19B—C18B	110.9 (17)
C24—C25—H25H	109.5	C20B—C19B—H19C	109.5
C24—C25—H25I	109.5	C20B—C19B—H19D	109.5
H25G—C25—H25H	109.5	C19B—C20B—H20C	108.5
H25G—C25—H25I	109.5	C19B—C20B—H20D	108.5
H25H—C25—H25I	109.5	H20C—C20B—H20D	107.5
C24—C26—H26G	109.5	C21B—C20B—C19B	115.1 (19)
C24—C26—H26H	109.5	C21B—C20B—H20C	108.5
C24—C26—H26I	109.5	C21B—C20B—H20D	108.5
H26G—C26—H26H	109.5	C20B—C21B—H21B	114.3
H26G—C26—H26I	109.5	C20B—C21B—C22B	118 (2)
H26H—C26—H26I	109.5	C20B—C21B—C24B	103.6 (19)
C8A—O1A—C1A	117.4 (4)	C22B—C21B—H21B	114.3
C3A—O2A—C2A	116.0 (3)	C22B—C21B—C24B	88.9 (17)
C11A—O3A—C12A	108.9 (3)	C24B—C21B—H21B	114.3
C15A—O4A—C16A	107.0 (4)	C21B—C22B—H22C	114.3
C16B—O4A—C15A	130.2 (12)	C21B—C22B—H22D	114.3
C10A—N1A—C9A	109.7 (3)	C21B—C22B—C23B	86.4 (17)
C10A—N1A—C13A	107.8 (3)	H22C—C22B—H22D	111.4
C10A—N1A—C14A	113.3 (3)	C23B—C22B—H22C	114.3
C13A—N1A—C9A	110.5 (3)	C23B—C22B—H22D	114.3
C13A—N1A—C14A	108.6 (3)	C18B—C23B—C22B	107 (2)
C14A—N1A—C9A	106.8 (3)	C18B—C23B—H23B	113.9
O1A—C1A—H1AA	109.5	C18B—C23B—C24B	117.9 (19)
O1A—C1A—H1AB	109.5	C22B—C23B—H23B	113.9
O1A—C1A—H1AC	109.5	C22B—C23B—C24B	87.5 (16)
H1AA—C1A—H1AB	109.5	C24B—C23B—H23B	113.9
H1AA—C1A—H1AC	109.5	C21B—C24B—C23B	82.8 (16)
H1AB—C1A—H1AC	109.5	C25B—C24B—C21B	108 (2)
O2A—C2A—H2AA	109.5	C25B—C24B—C23B	110 (2)
O2A—C2A—H2AB	109.5	C26B—C24B—C21B	124 (2)
O2A—C2A—H2AC	109.5	C26B—C24B—C23B	127 (2)
H2AA—C2A—H2AB	109.5	C26B—C24B—C25B	104 (2)
H2AA—C2A—H2AC	109.5	C24B—C25B—H25D	109.5
H2AB—C2A—H2AC	109.5	C24B—C25B—H25E	109.5
O2A—C3A—C8A	115.4 (4)	C24B—C25B—H25F	109.5
C4A—C3A—O2A	124.4 (4)	H25D—C25B—H25E	109.5
C4A—C3A—C8A	120.1 (4)	H25D—C25B—H25F	109.5
C3A—C4A—H4A	118.9	H25E—C25B—H25F	109.5
C3A—C4A—C5A	122.1 (4)	C24B—C26B—H26D	109.5
C5A—C4A—H4A	118.9	C24B—C26B—H26E	109.5
C4A—C5A—C9A	117.8 (4)	C24B—C26B—H26F	109.5
C6A—C5A—C4A	116.9 (4)	H26D—C26B—H26E	109.5
C6A—C5A—C9A	125.2 (4)	H26D—C26B—H26F	109.5
C5A—C6A—Br1A	121.5 (3)	H26E—C26B—H26F	109.5
C5A—C6A—C7A	121.8 (5)		
			
Br1—C6—C7—C8	174.4 (3)	C4A—C3A—C8A—O1A	179.5 (4)
O2—C3—C4—C5	−178.9 (4)	C4A—C3A—C8A—C7A	−1.7 (6)
O2—C3—C8—O1	−1.6 (5)	C4A—C5A—C6A—Br1A	173.9 (3)
O2—C3—C8—C7	179.4 (4)	C4A—C5A—C6A—C7A	−3.6 (6)
O3—C12—C13—N1	−59.8 (4)	C4A—C5A—C9A—N1A	87.2 (5)
O4—C16—C17—C18	−174.0 (4)	C5A—C6A—C7A—C8A	1.1 (7)
N1—C10—C11—O3	57.7 (4)	C6A—C5A—C9A—N1A	−96.9 (5)
N1—C14—C15—O4	−60.2 (5)	C6A—C7A—C8A—O1A	−179.6 (4)
C1—O1—C8—C3	−172.5 (4)	C6A—C7A—C8A—C3A	1.6 (6)
C1—O1—C8—C7	6.5 (6)	C8A—C3A—C4A—C5A	−1.0 (6)
C2—O2—C3—C4	8.2 (6)	C9A—N1A—C10A—C11A	−172.0 (3)
C2—O2—C3—C8	−172.6 (4)	C9A—N1A—C13A—C12A	170.7 (3)
C3—C4—C5—C6	−1.4 (6)	C9A—N1A—C14A—C15A	−81.9 (4)
C3—C4—C5—C9	−173.3 (4)	C9A—C5A—C6A—Br1A	−2.1 (6)
C4—C3—C8—O1	177.6 (4)	C9A—C5A—C6A—C7A	−179.6 (4)
C4—C3—C8—C7	−1.4 (6)	C10A—N1A—C9A—C5A	71.8 (4)
C4—C5—C6—Br1	−173.6 (3)	C10A—N1A—C13A—C12A	50.8 (4)
C4—C5—C6—C7	0.2 (6)	C10A—N1A—C14A—C15A	39.1 (4)
C4—C5—C9—N1	−88.9 (4)	C11A—O3A—C12A—C13A	63.1 (4)
C5—C6—C7—C8	0.2 (6)	C12A—O3A—C11A—C10A	−64.3 (4)
C6—C5—C9—N1	99.9 (5)	C13A—N1A—C9A—C5A	−47.0 (5)
C6—C7—C8—O1	−178.6 (4)	C13A—N1A—C10A—C11A	−51.6 (4)
C6—C7—C8—C3	0.3 (6)	C13A—N1A—C14A—C15A	158.9 (3)
C8—C3—C4—C5	2.0 (6)	C14A—N1A—C9A—C5A	−164.9 (4)
C9—N1—C10—C11	−170.5 (3)	C14A—N1A—C10A—C11A	68.7 (4)
C9—N1—C13—C12	171.9 (3)	C14A—N1A—C13A—C12A	−72.4 (4)
C9—N1—C14—C15	73.7 (5)	C15A—O4A—C16A—C17A	155.1 (5)
C9—C5—C6—Br1	−2.4 (5)	C15A—O4A—C16B—C17B	−172.9 (15)
C9—C5—C6—C7	171.4 (4)	C16A—O4A—C15A—C14A	177.1 (4)
C10—N1—C9—C5	46.2 (5)	C16A—C17A—C18A—C19A	−166.7 (6)
C10—N1—C13—C12	51.7 (4)	C16A—C17A—C18A—C23A	64.9 (8)
C10—N1—C14—C15	−167.0 (4)	C17A—C18A—C19A—C20A	−134.9 (8)
C11—O3—C12—C13	64.2 (4)	C17A—C18A—C23A—C22A	178.3 (6)
C12—O3—C11—C10	−63.6 (4)	C17A—C18A—C23A—C24A	83.7 (7)
C13—N1—C9—C5	−72.6 (4)	C18A—C19A—C20A—C21A	0.6 (12)
C13—N1—C10—C11	−50.5 (4)	C18A—C23A—C24A—C21A	82.5 (6)
C13—N1—C14—C15	−47.5 (5)	C18A—C23A—C24A—C25A	−166.4 (6)
C14—N1—C9—C5	164.5 (4)	C18A—C23A—C24A—C26A	−36.6 (9)
C14—N1—C10—C11	71.7 (4)	C19A—C18A—C23A—C22A	50.7 (8)
C14—N1—C13—C12	−68.1 (4)	C19A—C18A—C23A—C24A	−43.9 (8)
C15—O4—C16—C17	173.3 (4)	C19A—C20A—C21A—C22A	−47.4 (11)
C16—O4—C15—C14	−165.9 (4)	C19A—C20A—C21A—C24A	52.1 (11)
C16—C17—C18—C19	−66.3 (6)	C20A—C21A—C22A—C23A	84.5 (7)
C16—C17—C18—C23	165.1 (4)	C20A—C21A—C24A—C23A	−85.4 (7)
C17—C18—C19—C20	−135.3 (5)	C20A—C21A—C24A—C25A	165.6 (7)
C17—C18—C23—C22	−177.6 (5)	C20A—C21A—C24A—C26A	36.1 (10)
C17—C18—C23—C24	87.1 (6)	C21A—C22A—C23A—C18A	−86.6 (7)
C18—C19—C20—C21	3.9 (7)	C21A—C22A—C23A—C24A	27.6 (6)
C18—C23—C24—C21	80.7 (5)	C22A—C21A—C24A—C23A	27.9 (5)
C18—C23—C24—C25	−39.9 (8)	C22A—C21A—C24A—C25A	−81.1 (7)
C18—C23—C24—C26	−167.2 (5)	C22A—C21A—C24A—C26A	149.4 (7)
C19—C18—C23—C22	53.2 (6)	C22A—C23A—C24A—C21A	−26.4 (5)
C19—C18—C23—C24	−42.1 (6)	C22A—C23A—C24A—C25A	84.7 (6)
C19—C20—C21—C22	−48.3 (7)	C22A—C23A—C24A—C26A	−145.5 (7)
C19—C20—C21—C24	47.7 (7)	C23A—C18A—C19A—C20A	−4.0 (10)
C20—C21—C22—C23	85.2 (5)	C24A—C21A—C22A—C23A	−28.3 (5)
C20—C21—C24—C23	−82.7 (5)	C16B—O4A—C15A—C14A	174.9 (19)
C20—C21—C24—C25	40.2 (6)	C16B—C17B—C18B—C19B	−64 (3)
C20—C21—C24—C26	164.5 (5)	C16B—C17B—C18B—C23B	177 (2)
C21—C22—C23—C18	−87.5 (5)	C17B—C18B—C19B—C20B	−131 (2)
C21—C22—C23—C24	27.8 (4)	C17B—C18B—C23B—C22B	−179 (2)
C22—C21—C24—C23	27.7 (4)	C17B—C18B—C23B—C24B	85 (3)
C22—C21—C24—C25	150.6 (5)	C18B—C19B—C20B—C21B	−2 (3)
C22—C21—C24—C26	−85.0 (5)	C18B—C23B—C24B—C21B	80 (2)
C22—C23—C24—C21	−27.5 (5)	C18B—C23B—C24B—C25B	−173 (2)
C22—C23—C24—C25	−148.2 (6)	C18B—C23B—C24B—C26B	−47 (4)
C22—C23—C24—C26	84.6 (5)	C19B—C18B—C23B—C22B	64 (3)
C23—C18—C19—C20	−6.5 (7)	C19B—C18B—C23B—C24B	−32 (3)
C24—C21—C22—C23	−27.8 (4)	C19B—C20B—C21B—C22B	−36 (3)
Br1A—C6A—C7A—C8A	−176.6 (3)	C19B—C20B—C21B—C24B	60 (3)
O2A—C3A—C4A—C5A	177.4 (4)	C20B—C21B—C22B—C23B	76 (2)
O2A—C3A—C8A—O1A	0.9 (6)	C20B—C21B—C24B—C23B	−90.5 (19)
O2A—C3A—C8A—C7A	179.8 (4)	C20B—C21B—C24B—C25B	161 (2)
O3A—C12A—C13A—N1A	−57.7 (4)	C20B—C21B—C24B—C26B	40 (3)
O4A—C16A—C17A—C18A	71.0 (7)	C21B—C22B—C23B—C18B	−90 (2)
O4A—C16B—C17B—C18B	−175 (2)	C21B—C22B—C23B—C24B	28.6 (17)
N1A—C10A—C11A—O3A	59.9 (4)	C22B—C21B—C24B—C23B	28.0 (17)
N1A—C14A—C15A—O4A	55.5 (5)	C22B—C21B—C24B—C25B	−80 (2)
C1A—O1A—C8A—C3A	−179.5 (4)	C22B—C21B—C24B—C26B	158 (3)
C1A—O1A—C8A—C7A	1.7 (6)	C22B—C23B—C24B—C21B	−27.3 (16)
C2A—O2A—C3A—C4A	−4.9 (6)	C22B—C23B—C24B—C25B	80 (2)
C2A—O2A—C3A—C8A	173.6 (4)	C22B—C23B—C24B—C26B	−154 (3)
C3A—C4A—C5A—C6A	3.6 (6)	C23B—C18B—C19B—C20B	−12 (3)
C3A—C4A—C5A—C9A	179.9 (4)	C24B—C21B—C22B—C23B	−28.5 (17)

## References

[bb1] Bruker (2016). *SAINT.* Bruker AXS, Inc., Madison, Wisconsin, USA.

[bb2] Bruker (2023). *APEX5*. Bruker AXS, Inc., Madison, Wisconsin, USA.

[bb3] Burnett, M. N. & Johnson, C. K. (1996). *ORTEPIII*. Report ORNL-6895. Oak Ridge National Laboratory, Tennessee, USA.

[bb4] Cremer, D. & Pople, J. A. (1975). *J. Am. Chem. Soc.* **97**, 1354–1358.

[bb5] Dolomanov, O. V., Bourhis, L. J., Gildea, R. J., Howard, J. A. K. & Puschmann, H. (2009). *J. Appl. Cryst.* **42**, 339–341.

[bb6] Krause, L., Herbst-Irmer, R., Sheldrick, G. M. & Stalke, D. (2015). *J. Appl. Cryst.* **48**, 3–10. 10.1107/S1600576714022985 PMC4453166 26089746

[bb7] Macrae, C. F., Sovago, I., Cottrell, S. J., Galek, P. T. A., McCabe, P., Pidcock, E., Platings, M., Shields, G. P., Stevens, J. S., Towler, M. & Wood, P. A. (2020). *J. Appl. Cryst.* **53**, 226–235. 10.1107/S1600576719014092 PMC6998782 32047413

[bb8] Sheldrick, G. M. (2015*a*). *Acta Cryst.* A**71**, 3–8.

[bb9] Sheldrick, G. M. (2015*b*). *Acta Cryst.* C**71**, 3–8.

[bb10] Wikipedia (2023). Pinaverium bromide, https://en. wikipedia. org/wiki/Pinaverium_bromide (accessed 30 June 2024).

[bb11] Zheng, L., Lai, Y., Lu, W., Li, B., Fan, H., Yan, Z., Gong, Ch., Wan, X., Wu, J., Huang, D., Wang, Y., Mei, Y., Li, Z., Jiang, Z., Liu, X., Ye, J., Yang, Y., Huang, H. & Xiao, J. (2015). *Clin. Gastroenterol. Hepatol.* **13**, 1285–1292.e1. 10.1016/j.cgh.2015.01.015 25632806

